# Deterioration of muscle force and contractile characteristics are early pathological events in spinal and bulbar muscular atrophy mice

**DOI:** 10.1242/dmm.042424

**Published:** 2020-05-26

**Authors:** Anna L. Gray, Leonette Annan, James R. T. Dick, Albert R. La Spada, Michael G. Hanna, Linda Greensmith, Bilal Malik

**Affiliations:** 1 Department of Neuromuscular Diseases, UCL Queen Square Institute of Neurology, Queen Square, London WC1N 3BG, UK; 2 Department of Neurology, Duke University School of Medicine, Durham, NC 27710, USA; 3 Department of Neurobiology, Duke University School of Medicine, Durham, NC 27710, USA; 4 Department of Cell Biology, Duke University School of Medicine, Durham, NC 27710, USA; 5 Duke Center for Neurodegeneration and Neurotherapeutics, Duke University School of Medicine, Durham, NC 27710, USA; 6 Department of Neuroscience, University of California, San Diego, La Jolla, CA 92093, USA; 7 UCL MRC International Centre for Genomic Medicine in Neuromuscular Diseases, UCL Queen Square Institute of Neurology, Queen Square, London WC1N 3BG, UK

**Keywords:** SBMA, Androgen receptor, Muscle, Myopathy, Neuromuscular disease, Spinal and bulbar muscular atrophy

## Abstract

Spinal and bulbar muscular atrophy (SBMA), also known as Kennedy's Disease, is a late-onset X-linked progressive neuromuscular disease, which predominantly affects males. The pathological hallmarks of the disease are selective loss of spinal and bulbar motor neurons, accompanied by weakness, atrophy and fasciculations of bulbar and limb muscles. SBMA is caused by a CAG repeat expansion in the gene that encodes the androgen receptor (AR) protein. Disease manifestation is androgen dependent and results principally from a toxic gain of AR function. There are currently no effective treatments for this debilitating disease. It is important to understand the course of the disease in order to target therapeutics to key pathological stages. This is especially relevant in disorders such as SBMA, for which disease can be identified before symptom onset, through family history and genetic testing. To fully characterise the role of muscle in SBMA, we undertook a longitudinal physiological and histological characterisation of disease progression in the AR100 mouse model of SBMA. Our results show that the disease first manifests in skeletal muscle, before any motor neuron degeneration, which only occurs in late-stage disease. These findings reveal that alterations in muscle function, including reduced muscle force and changes in contractile characteristics, are early pathological events in SBMA mice and suggest that muscle-targeted therapeutics may be effective in SBMA.

This article has an associated First Person interview with the first author of the paper.

## Abstract (precis)

**Summary:** Research on the AR100 mouse model of spinal and bulbarmuscular atrophy shows that disease manifests first in skeletal muscle, before motor neuron degeneration, which only occurs in the late stage of disease.

## INTRODUCTION

Spinal and bulbar muscular atrophy (SBMA), also known as Kennedy's Disease, is a late-onset X-linked progressive neuromuscular disease, which predominantly affects males. Clinically, the disease is characterised by loss of bulbar and spinal motor neurons with atrophy, fasciculations and weakness of bulbar, facial and limb muscles (Kennedy et al., 1968; Rhodes et al., 2009). SBMA is caused by a trinucleotide CAG repeat expansion in the gene that encodes the androgen receptor (AR) protein (La Spada et al., 1991). Disease manifestation is androgen dependent and results mainly from a toxic gain of AR function. *AR* is localised to position q11-q12 on the long arm of the X chromosome (Brown et al., 1989; Lubahn et al., 1988). The gene consists of eight exons constituting around 919 amino acids, which encode several functional domains (Poletti, 2004; Werner and Holterhus, 2014). The SBMA-causing CAG repeat expansion was identified within exon 1 and encodes a polyglutamine (polyQ) tract in the mature protein (La Spada et al., 1991). In healthy individuals the polymorphic CAG repeat tract ranges between nine and 36 repeats, and CAG repeat lengths greater than 37 are associated with SBMA (Fischbeck, 1997). As yet there are no effective treatments or disease-modifying therapies for the disease.

In addition to its classification as a motor neuron disease (MND) and neuromuscular disorder, SBMA also belongs to a group of polyglutamine or polyQ expansion diseases (Poletti, 2004). These nine genetically inherited disorders include Huntington's disease, dentatorubral-pallidoluysian atrophy and spinocerebellar ataxia types 1, 2, 3, 6, 7 and 17. The mutant proteins are expressed in numerous tissues and share no homology other than the expanded polyQ tracts, yet all result in selective neuronal death (Adegbuyiro et al., 2017). Despite the clinical heterogeneity of the polyQ expansion diseases, owing to the function and distribution of the affected proteins, they still share similar underlying molecular mechanisms involved in disease pathogenesis (Palazzolo et al., 2008).

Although expression of the expanded polyQ-AR is known to be causative for SBMA and the protein is expressed ubiquitously (La Spada et al., 1991), the selectivity of bulbar and lower motor neuron loss as well as degeneration of muscle is still poorly understood. Significantly, it was shown that ligand-dependent translocation of the polyQ-AR to the nucleus is necessary for disease pathogenesis (Takeyama et al., 2002; Walcott and Merry, 2002). Furthermore, nuclear accumulation (Adachi et al., 2005; Takeyama et al., 2002; Walcott and Merry, 2002) altered conformation and impaired clearance of the aberrant protein (Cortes et al., 2014b; Montie et al., 2009; Rusmini et al., 2011), as well as endoplasmic reticulum (ER) stress (Montague et al., 2014) and transcriptional dysregulation (Iida et al., 2014; Malik et al., 2013, 2019; Rocchi et al., 2016; Sopher et al., 2004) are all thought to be contributing factors. There may also be a slight loss of function of AR contributing to SBMA pathogenesis (Thomas et al., 2006). However, motor impairment has not been observed in AR knockout mice (Yeh et al., 2002) or in severe testicular feminisation patients lacking AR function (Batista et al., 2018). Interestingly, recent reports have shown that muscle may play a more prominent part in disease than previously thought and indeed may be a key site of AR toxicity and the development of pathology in SBMA (Cortes et al., 2014a; Lieberman et al., 2014; Milioto et al., 2017).

In this study, we set out to fully characterise the physiological basis of the muscle dysfunction evident in the AR100 mouse model of SBMA. AR100 transgenic mice carry 100 polyQ-encoding CAG repeats in the human *AR* gene and develop a progressive neuromuscular phenotype with accompanying lower motor neuron degeneration and muscle atrophy, which closely recapitulates the human disease (Malik et al., 2011, 2013; Sopher et al., 2004). Muscle is an attractive target for therapeutic intervention as, compared to motor neurons in the CNS, it is relatively accessible. Therefore, it is essential to fully understand muscle dysfunction in SBMA. Furthermore, it is important to establish the development of pathology and appreciate the progression and course of the disease, which will ultimately assist in the targeting of therapeutics to key disease stages. This is especially important in hereditary disorders such as SBMA, for which disease can be identified before symptom onset, through family history and genetic testing.

Our results show that, in the AR100 mouse model of SBMA, although considered a motor neuron disease, symptoms first manifest in hindlimb skeletal muscle, before any motor neuron degeneration, which only occurs in late-stage disease. These findings confirm that muscle plays a key role in disease pathogenesis in SBMA and may indeed be the primary site of AR toxicity, suggesting that muscle-targeted therapeutics may be particularly effective in SBMA.

## RESULTS

### Deficits in hindlimb muscle force and muscle fatigue characteristics occur early in disease in SBMA mice

To understand the course of disease and the development of pathology in muscle in SBMA, we characterised male SBMA AR100 YAC transgenic mice carrying 100 CAG repeats within the *AR* gene (Malik et al., 2013; Sopher et al., 2004). Although behavioural analysis has previously been used to determine a disease phenotype (Sopher et al., 2004) and the neuromuscular deficit has been examined at a late stage of disease (Malik et al., 2011, 2013), a thorough characterisation of disease progression including longitudinal *in vivo* physiological isometric muscle force testing and examination of muscle contractility has never been undertaken in mouse models of SBMA, including AR100 mice. Therefore, in this study, to fully characterise the progression of muscle deficits over the course of disease, we undertook an *in vivo* physiological examination of hindlimb muscle function accompanied by histopathological analysis of muscle and spinal cord of male AR100 mice at different ages (3, 6, 12 and 18 months). This may help to identify key disease stages in SBMA male AR100 mice and relative contribution of muscle and spinal cord pathology to the disease phenotype.

**Fig. 1.**
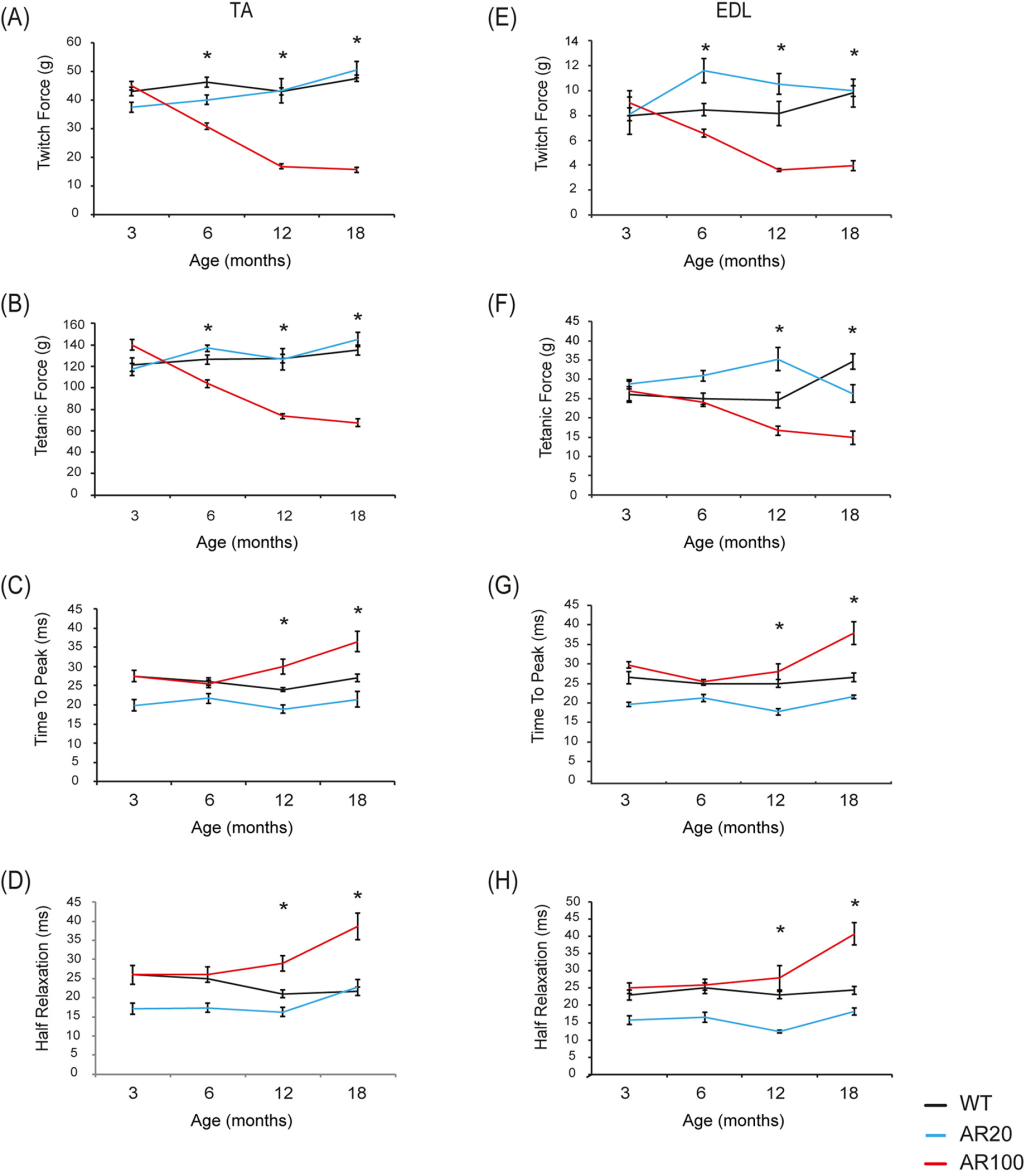
**Muscle** **force and contractile characteristics of fast-twitch muscles deteriorate over the course of disease in SBMA mice.** (A,B) In AR100 mice, there was a clear progressive degeneration in both maximal twitch (A) and maximal tetanic force (B), compared with WT and control AR20 mice. The disease-related decline in twitch and tetanic tension in AR100 mice was significant from 6 months of age. At 12 months, the muscle force deficits plateaued, with little further decline thereafter. (C,D) The contraction/relaxation properties, including TTP (C) and ½RT (D) were prolonged in AR100 mice as disease progressed. This decline happened later than the decline in force and only reached statistical significance at 18 months of age in AR100 mice compared to WT and AR20 mice. (E) In AR100 mice, there was a progressive decline in maximal twitch force throughout the disease course, significant from 6 months of age. (F) The decline in maximal tetanic force was significantly different from WT and AR20 mice from 12 months of age. (G,H) The contraction/relaxation properties, including TTP (G) and ½RT (H) were prolonged in AR100 mice relative to WT and AR20 mice. This change happened later in disease than the muscle force reduction, with the only significant differences evident at the late stage of disease (18 months). The experiments were terminal and therefore the data points represent different mice examined at each time point. Statistical analysis was performed using one-way ANOVA followed by the Student–Newman–Keuls and Tukey's Honestly Significantly Different post hoc tests (*n*≥5 animals, *P<0.05). Error bars represent s.e.m.

By using *in vivo* muscle isometric force measurement in young male mice at 3 months of age, we showed that there was no difference in maximum twitch or tetanic muscle force between hindlimb tibialis anterior (TA) muscles of AR100 mice and wild-type (WT) and control AR20 mice (Fig. 1A,B). However, by 6 months of age, we observed a clear physiological deficit in the fast-twitch TA hindlimb muscle in AR100 mice, with a significant reduction of ∼30% in maximal twitch muscle force, as well as a decrease of ∼18% in maximal tetanic force compared to control WT mice (Fig. 1A,B). By 12 months of age, there was a further decline in muscle force, at which point maximal twitch force in AR100 mice was reduced by 61% and maximal tetanic force was reduced by 42% (Fig. 1A,B) compared to WT mice. There was no significant difference in twitch or tetanic force of TA in WT and control non-pathogenic AR20 mice.

We also examined the contractile characteristics of the TA fast-twitch muscle. Normally, fast-twitch muscles such as TA contract and relax rapidly, with the time taken to reach peak (maximum) contraction (TTP) and to relax (assessed as the time taken to reach half of the maximum force; ½RT) being rapid. At 3 months of age, there was no change in the contractile characteristics of TA muscles of AR100 mice, with the TTP (Fig. 1C) and ½RT similar across all three strains of mice (Fig. 1D). From 6 months of age, there was a progressive slowing of the contractile characteristics of TA muscles of AR100 mice, with an increase in TTP and ½RT, with AR100 muscle significantly different from WT and AR20 TA at 12 and 18 months of age (Fig. 1C,D).

We next studied muscle force properties in the fast-twitch extensor digitorum longus (EDL) muscle. EDL muscles of AR100 mice demonstrated a progressive decline in force and contractile characteristics over the disease course, similar to that observed in TA muscles. The maximal twitch force was significantly reduced by 6 months of age (Fig. 1E) and, although reduced, maximal tetanic force was not statistically different from WT values until 12 months of age (Fig. 1F). The contractile properties of the EDL muscle were also altered in the latter stages of the disease, with a significant increase in TTP (Fig. 1G) and ½RT (Fig. 1H) of EDL in AR100 mice by 18 months.

In the slow-twitch soleus muscle, changes in both muscle force and contractile characteristics displayed a different pattern to that observed in TA and EDL (Fig. S1), with a reduction in muscle force later than the fast-twitch muscles. Therefore, at 12 months, there was significant decrease in twitch force and tetanic force of soleus in AR100 mice (Fig. S1). Furthermore, there were few, if any, changes in the contractile characteristics of soleus of AR100 mice, with a significant increase in TTP only detected at 12 months (Fig. S1C,D). This is perhaps not surprising, as soleus is inherently a slow-twitch muscle.

Therefore, muscle force deterioration was evident in AR100 mice by 6 months of age in both TA and EDL hindlimb muscles, with the loss in muscle force near maximal by 12 months of age and with little deterioration observed later in disease. However, the loss of contractile characteristics was a property of late-phase disease for both TA and EDL muscles, with progressive slowing in the speed of contraction and relaxation, although these changes only became significant at later stages of disease (12 to 18 months).

**Fig. 2.**
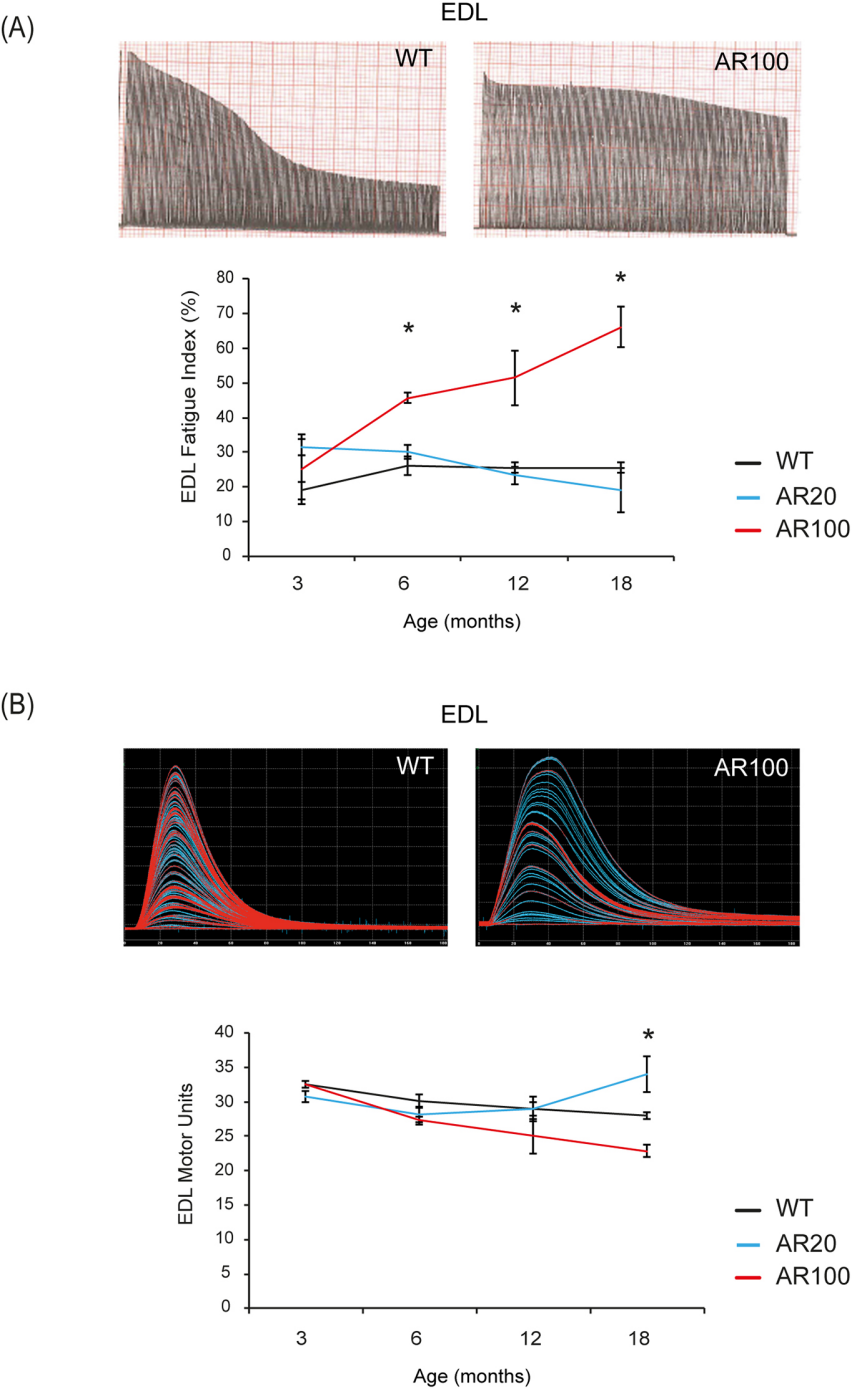
**Changes in innervation and fatigue characteristics of hindlimb** **muscle in SBMA mice.** (A) Representative EDL fatigue traces from WT and AR100 are shown. The fatigue index (FI) values of EDL muscles in AR100 mice were significantly higher than WT and AR20 mice mice at 6, 12 and 18 months of age. EDL muscles in AR100 mice became more resistant to fatigue as disease progressed. (B) EDL motor unit number was the same in WT and AR100 mice at 3 months of age. From 6 months of age, motor unit number was reduced in AR100 mice compared to WT mice and there was a gradual decline with ageing. Statistical analysis was performed using one-way ANOVA followed by the Student–Newman–Keuls and Tukey's Honestly Significantly Different post hoc tests (*n*≥5 animals, *P<0.05). Error bars represent s.e.m.

A characteristic feature of fast-twitch muscles such as EDL is that they normally fatigue rapidly and are unable to maintain force when repeatedly stimulated. This characteristic can be assessed by calculating a fatigue index (FI), which is a measure of the force produced by a muscle at the end of a 3 min period of stimulation, relative to the force produced at the start. As can be seen in Fig. 2, there was no change in the fatigue characteristics of EDL muscles of AR100 mice at 3 months of age, but thereafter, EDL muscles of AR100 mice became progressively more resistant to fatigue, with a clear decrease in the fatigability of EDL of AR100 mice (Fig. 2A). Thus, by 6 months of age, the EDL muscle in AR100 mice became more fatigue resistant, with an increased FI of nearly 43% compared to WT mice [AR100 FI: 45.69%±1.5 (mean±s.e.m.), *n*=14, versus WT mice FI: 26.02%±2.7, *n*=9; *P*<0.05]. In contrast, the fatigability of the soleus muscle, normally a fatigue-resistant muscle, was not altered in AR100 mice at any age examined with the fatigue index, similar to that of WT soleus (Fig. S1E).

### Early loss of functional motor units in hindlimb muscles of SBMA mice

The number of functional motor units innervating the EDL and soleus muscles was determined by gradually increasing the strength of the stimulus applied to the sciatic nerve, allowing incremental recruitment of motor axons with increasing stimulus thresholds, resulting in stepwise increments in twitch tension which can be counted to estimate the number of functional motor units (Fig. 2B). At 3 months of age, there was no difference in the number of motor units innervating EDL muscle between WT, AR20 and AR100 mice. By 6 months, we found a 10% reduction in the number of motor units innervating EDL muscles of AR100 mice compared with WT mice (AR100 mice: 27.27±0.6, *n*=11, versus WT mice: 30.11±1, *n*=9; *P*<0.05). However, there was no difference between AR20 and AR100 mice at that age. By 18 months, there was a 20% reduction in motor unit survival in EDL muscles of AR100 mice (22.83±0.88, *n*=12; *P*<0.05), which was significantly reduced compared to WT mice (28±0.43, *n*=18) and AR20 mice (34±2.6, *n*=10) (Fig. 2B). Similar to the muscle force and contractile characteristics, there were no deficits in motor unit survival in the soleus muscle, so that the number of functional motor units innervating the soleus muscle in WT and AR100 mice was equivalent at 6, 12 and 18 months of age (Fig. S1F).

### Deficits in hindlimb muscle force is accompanied by loss of body weight and hindlimb muscle atrophy

**Fig. 3.**
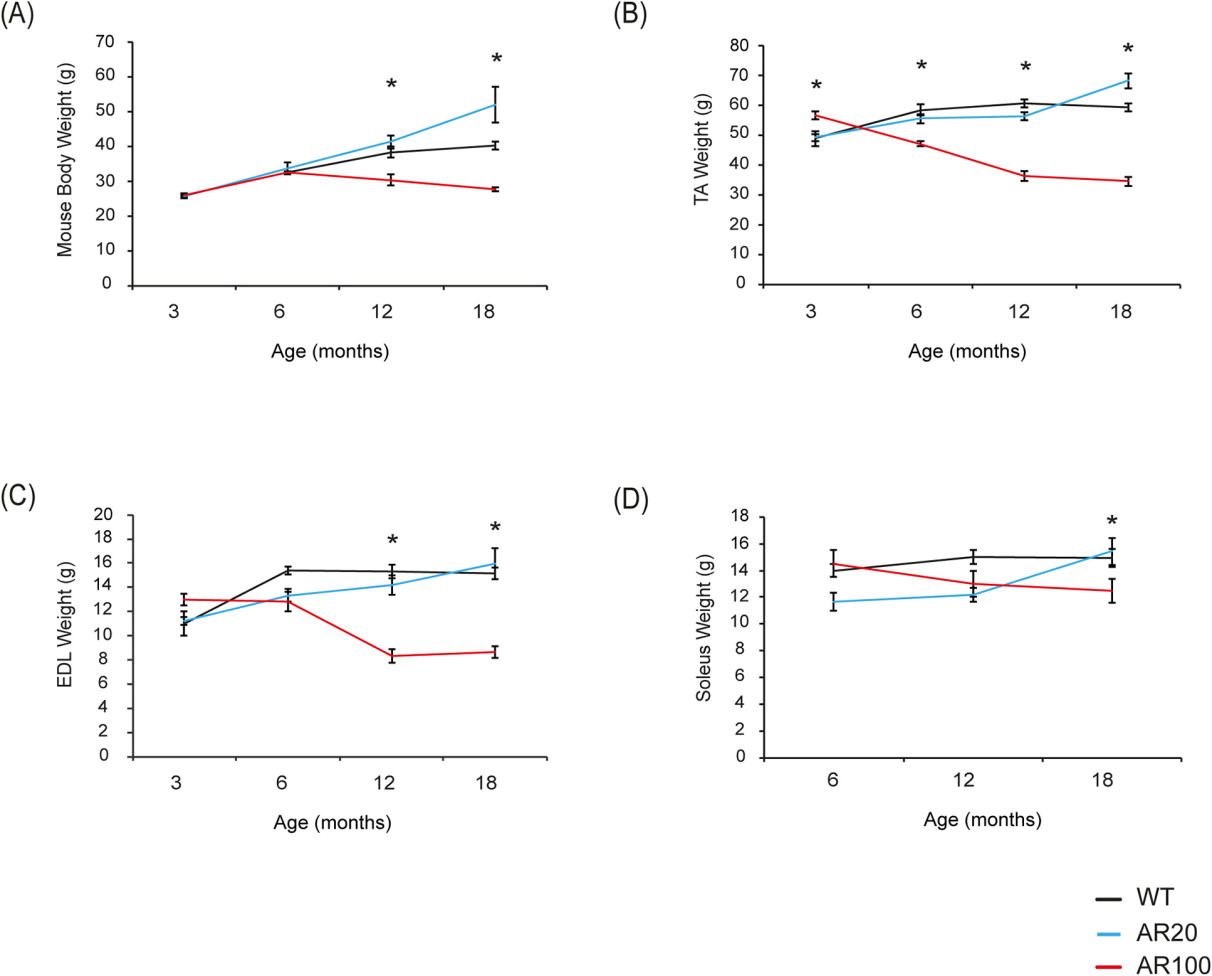
**Loss of body and hindlimb muscle weight during disease progression in SBMA mice.** (A) Body weight did not differ between WT and AR100 mice at 3 or 6 months of age. However, at both 12 and 18 months, AR100 mice weighed significantly less than WT and AR20 mice. (B) TA muscle weight was slightly higher in AR100 mice at 3 months. However, at 6, 12 and 18 months of age there was a significant reduction in TA muscle weight in AR100 mice compared to WT control mice. (C) There was no difference in EDL muscle weight between AR100 and WT and AR20 mice at 3 months. At 6, 12 and 18 months, however, the weights of EDL muscles in AR100 mice were considerably less than those of WT and AR20 mice. (D) Soleus muscle weights did not alter considerably over the course of disease; however, at the 18 month time point the difference just reached the threshold for statistical significance. Statistical analysis was performed using repeated measure or one-way ANOVA followed by the Student–Newman–Keuls and Tukey's Honestly Significantly Different post hoc tests (*n*≥5 animals, *P<0.05). Error bars represent s.e.m.

The body weight of SBMA mice was recorded as an indicator of disease progression. At 3 months of age, the AR100 mice were slightly heavier than the WT and AR20 mice (Fig. 3A). The body weight of WT and AR20 control mice progressively increased with age and at 6 months the body weight of WT, AR20 and AR100 mice was comparable (Fig. 3A). Thereafter, the body weight of AR100 mice declined progressively, and AR100 mice weighed significantly less than WT and AR20 mice at 12 and 18 months of age (12 months: *P*<0.05; 18 months: *P*<0.05; Fig. 3A).

We also determined the weight of the hindlimb muscles. At 3 months of age, TA in AR100 mice weighed significantly more than WT and AR20. There were no significant differences in the weight of EDL of AR100 mice at 3 months. However, by 6 months of age TA muscles weighed significantly less in AR100 mice than those of control WT and AR20 mice, and declined further by 12 months, with no additional deterioration between 12 and 18 months (Fig. 3B). The EDL in AR100 weighed less at 12 months than control WT and AR20 mice, with little decrease in weight after that age (Fig. 3C). In contrast, a significant reduction in the weight of the AR100 soleus muscle was only observed at late stage of disease (18 months: *P*<0.05, Fig. 3D).

### Histopathological changes in muscle during disease progression in SBMA mice

**Fig. 4.**
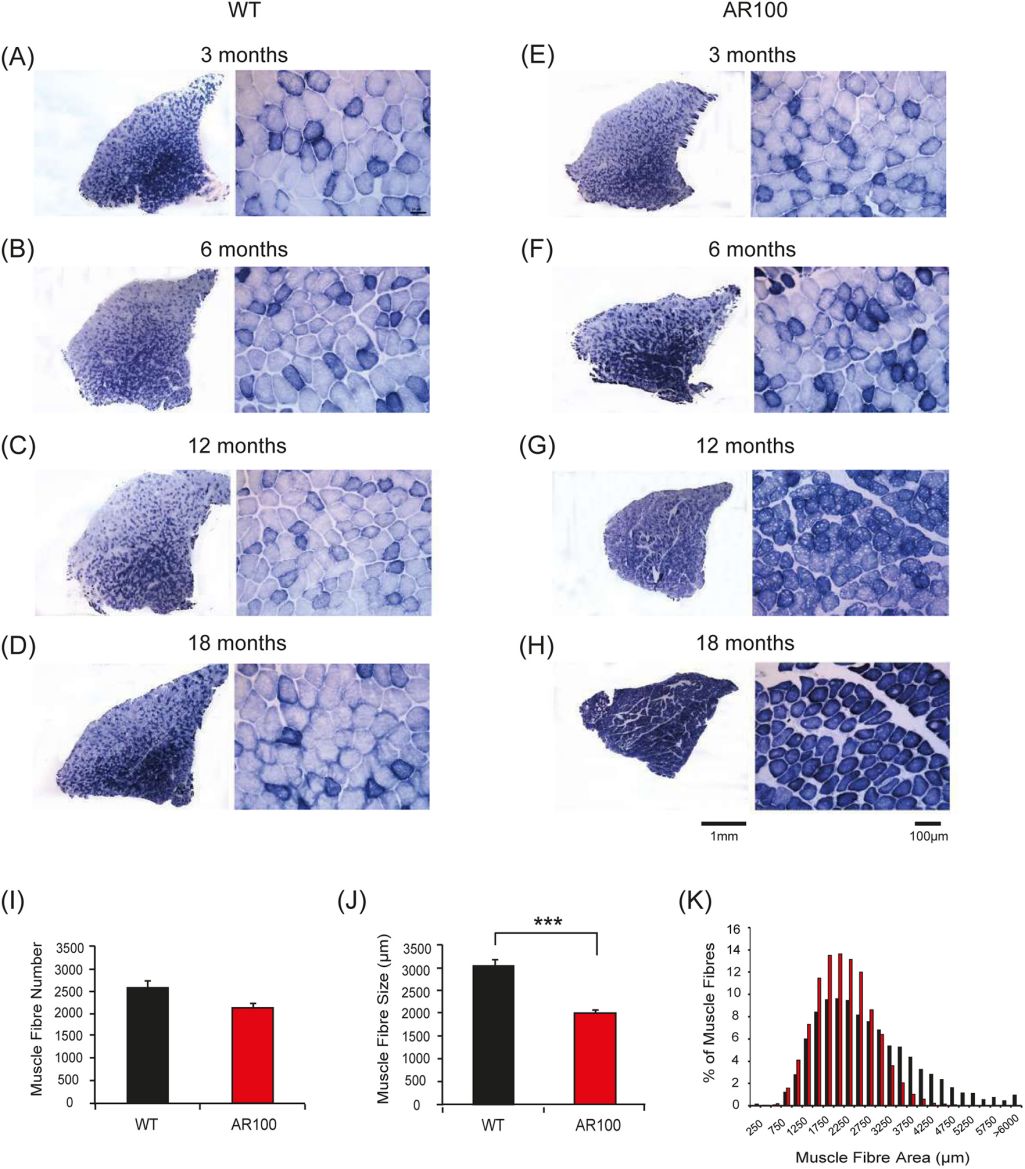
**Increase in oxidative capacity of TA muscle in SBMA mice with disease progression.** (A-H) Transverse TA muscle sections of WT (A-D) and AR100 (E-H) mice were stained for the mitochondrial respiratory enzyme SDH. (A-D) Muscle sections from WT mice at 3, 6, 12 and 18 months displayed a typical staining pattern, with intensely stained, highly oxidative fibres in the middle, and a characteristic mosaic pattern, representative of less oxidative, fast-twitch fibres in the outer region. (E) TA muscle of AR100 mice at 3 months of age was not different from that of WT mice. (F) At 6 months however, the first signs of muscle pathology were evident in AR100 mice. There appeared to be a slight increase in the number of intensely stained, oxidative fibres and evidence of muscle fibre grouping. (G) At 12 months of age, muscle fibre atrophy was clear and the number of intensely stained fibres was dramatically increased. (H) By 18 months of age the majority of fast-twitch fibres in AR100 TA muscle had been transformed to that of a slow-twitch, highly oxidative phenotype. Fibres were atrophied in comparison to those in WT TA muscles and muscle fibres were less densely packed, with increased space between them. (I) The number of muscle fibres present in AR100 mice was reduced but not significantly different to WT at 18 months of age. (J) At this late disease stage, the mean fibre area in TA of AR100 was significantly less than age-matched WT mice. (K) There was a clear reduction in larger size fibres accompanied by an increase in the number of smaller fibres. Statistical analysis was performed using Student's *t*-test (two-tailed) (*n*≥5 animals, ***P<0.001). Error bars represent s.e.m.

At the end of each acute physiology experiment, TA muscles were removed for histopathological analysis. Muscle sections were stained for succinate dehydrogenase (SDH), a mitochondrial membrane-bound respiratory enzyme, as a marker of oxidative capacity (Fig. 4). TA muscles are fast-twitch type II muscles and display a characteristic staining pattern, in which a central intensely stained oxidative core of fibres is surrounded by an outer region of largely glycolytic fibres which stain lightly for SDH, revealing a characteristic mosaic pattern of SDH staining in WT mice (Fig. 4A-D). This phenotype was not altered in TA muscle of AR100 mice at 3 months of age (Fig. 4E). However, by 6 months of age, the first signs of muscle pathology emerged, with an increase in the number of intensely stained (oxidative) fibres and evidence of muscle fibre type grouping of fibres of similar SDH staining intensity, rather than the more typical mosaic-like pattern of staining seen in WT TA (Fig. 4F). Importantly, this is the same stage of disease at which muscle atrophy and the first deficits in muscle force were detected. By 12 months of age, there was a considerable increase in the number and grouping of fibres that stained intensely for SDH in TA muscle of AR100 mice (Fig. 4G), with evidence of muscle fibre atrophy. By late stage of disease (18 months), the majority of TA fibres stained intensely for SDH, suggesting a dramatic shift in the muscle phenotype from a fast-twitch muscle to a slow-twitch muscle (Fig. 4H). Substantial atrophy of muscle fibres was evident at this stage of disease and the fibres were much less densely packed. Although the overall number of muscle fibres was not significantly reduced in AR100 mice compared to WT mice at 18 months of age (Fig. 4I), the mean fibre area in TA of AR100 (2166.4 μm²±69.8, *n*=3) was significantly less than age-matched WT mice (3023.2 μm²±123.1, *n*=3; *P*=0.004; Fig. 4J). By 18 months, there was a clear reduction in the number of larger size fibres and an increase in the number of smaller fibres (Fig. 4K).

**Fig. 5.**
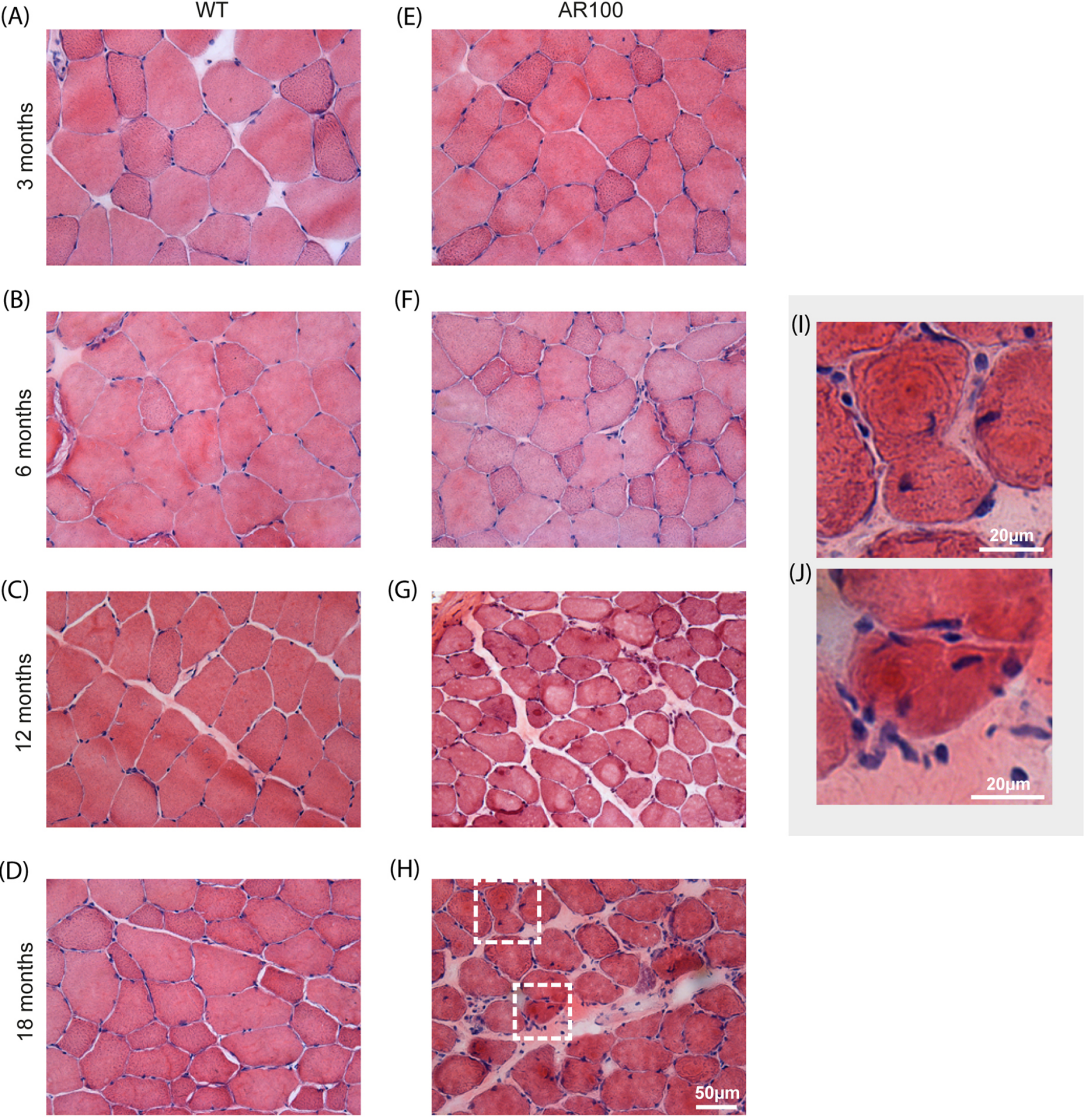
**H&E staining reveals extensive muscle pathology in aged SBMA mice.** (A-D) Transverse TA muscle sections from WT mice at 3, 6, 12 and 18 months displayed a typical staining pattern. Fibres were polygonal in shape, with non-fragmented sarcoplasm and peripheral nuclei. (E) AR100 TA muscles at 3 months of age were very similar to WT TA muscles. (F) At the 6-month time point little obvious morphological change was evident. (G,H) At 12 (G) and 18 (H) months, muscle fibres appeared atrophied, with frequent centralised nuclei. (I,J) High-magnification images of boxed areas in panel H, showing single muscle fibres from AR100 mice at 18 months of age.

Haematoxylin and Eosin (H&E) staining of WT TA muscle demonstrated a typical staining pattern at all ages, with consistent cytoplasmic staining in fibres, surrounded by multiple peripheral nuclei (Fig. 5A-D). A similar pattern of H&E staining was observed in AR100 TA muscle at 3 months (Fig. 5E) and 6 months (Fig. 5F) of age. However, by 12 months, TA muscle of AR100 mice showed considerable atrophy, with less densely packed fibres and frequent centralised nuclei (Fig. 5G). A similar pattern was observed at 18 months (Fig. 5H). Triangular, angulated fibres (typical of neurogenic denervation atrophy) were present by this stage, along with numerous centralised nuclei, most likely representing myogenic changes and muscle fibres attempting to regenerate (Fig. 5I,J)

We next stained fast-twitch TA fibres of 12-18 month AR100 mice for ubiquitin. No significant ubiquitin-positive staining was observed in WT muscle at any age examined (Fig. S2A-D) or in AR100 mice at 3 months of age (Fig. S2E). However, by 6 months, ubiquitin-positive staining was detected in TA of AR100 mice, albeit infrequently, usually localised to the outer rim of the sarcoplasm, adjacent to peripheral nuclei (Fig. S2F). Ubiquitin immunoreactivity increased as disease progressed so, by 12 months, ubiquitin staining was frequently observed within fibres in the outer, highly glycolytic region of the TA muscle (Fig. S2G), a pattern that was maintained at 18 months (Fig. S2H). No ubiquitin immunoreactivity was observed in the slow-twitch soleus muscle at any age examined (Fig. S2I).

As SDH staining suggested that fibre type grouping may occur in fast-twitch muscles during disease progression in AR100 mice, we also examined the pattern of expression of myosin heavy chain (MyHC) isoforms in order to characterise the fibre type composition of TA muscle (Bloemberg and Quadrilatero, 2012) (Fig. S3). Antibodies that recognise MyHC elements specific to slow-twitch oxidative (type IIA) and fast-twitch glycolytic (type IIB) fibres were used. At 6 months of age there was a slight increase in the proportion of type IIA fibres in AR100 TA compared to WT TA (Fig. S3A,B). By 12 months of age, type 2A muscle fibre composition was further increased in AR100 mice (Fig. S3C,D). As these features are associated with muscle denervation and reinnervation, it is likely that there is a neurogenic component to the muscle changes that occur in AR100 mice, although these deficits only manifest later in disease progression and appear to occur later than the primary effects on muscle force.

EDL muscles of WT, AR20 and AR100 mice were also analysed for signs of denervation, at different stages of disease progression. Longitudinal sections of EDL were stained with α-bungarotoxin, which labels the postsynaptic acetyl choline receptor, and choline acetyl transferase (ChAT), a marker of motor axons (Fig. S4). At 3 months of age, there was no significant difference in any of these parameters. By 6 months of age there was also no change in the number of denervated fibres between EDL from WT (12.3%±2.9, *n*=5), AR20 (11.0%±3.2, *n*=5) and AR100 mice (18.5%±4.2, *n*=7, ANOVA; *P*=0.321). By 18 months, there was a suggestion that denervation may occur. The expression of Nogo-A, a marker of muscle denervation (Bros-Facer et al., 2014), was also assessed in the TA muscle of AR100 mice (Fig. S5). At 3 and 6 months of age there was no difference between WT and AR100 muscle Nogo-A expression. By 12 months of age there was an increase in Nogo-A immunoreactivity in AR100 TA muscle, which was maintained in the muscles of 18-month-old mice. These findings confirm the fibre type grouping/switching results, suggesting that muscle denervation in TA of AR100 mice is a phenomena of late stage disease after the onset of muscle force deficits.

### Motor neuron survival

In order to establish the relationship between the development of muscle deficits in AR100 mice and motor neuron degeneration, we examined the survival of motor neurons in the sciatic motor pool of the spinal cord. We have previously shown that by late stage of disease, at 18 months, 40% of motor neurons in the sciatic motor pool have died in AR100 mice (Malik et al., 2011, 2013). Examination of motor neuron survival at earlier stages of the disease, at the age when muscle deficits first appeared, revealed that no motor neuron degeneration occured in AR100 mice at 6 months of age (Fig. S6), the age at which muscle force deficits are first evident, and when a 10% reduction in motor unit survival was observed in EDL muscles of AR100 mice compared to WT mice. Even by 12 months there was only a slight, but non-significant, loss of motor neurons in AR100 mice, despite the very significant pathology observed in fast-twitch muscles at this stage.

Taken together, these findings suggest that the significant reduction in muscle force observed in fast-twitch muscles of AR100 mice at as early as 6 months of age occurs in the absence of any motor neuron degeneration, indicative of a primary muscle deficit in the AR100 mouse model of SBMA. This suggests that the loss of motor units in the EDL muscles of 6-month-old AR100 mice, and changes associated with denervation/reinnervation (as reflected by muscle fibre type grouping), are not because of significant motor neuron degeneration, which only occurs in late stage of disease, between 12 and 18 months of age in AR100 mice.

## DISCUSSION

In this study, a comprehensive longitudinal pathophysiological characterisation of the AR100 mouse model of SBMA was undertaken in order to characterise the course of disease and the role of muscle in disease pathogenesis. A detailed understanding of the rate and sequence of events in disease pathogenesis is essential for the effective preclinical screening of novel therapeutic agents. This is especially relevant in SBMA, a disease with a known genetic cause for which therapeutic intervention can be targeted to early disease stages, before manifestation of symptoms. In this study, we provide compelling evidence that SBMA AR100 mice develop a progressive neuromuscular disorder driven by polyQ-AR, with the earliest pathology evident in fast-twitch hindlimb muscle. The early disease manifestation in hindlimb skeletal muscle occurs before any motor neuron degeneration, which only appears later in the disease process. Importantly, peripheral tissues such as muscle present an attractive target for therapy, as they are more accessible than CNS targets. Therefore, these results suggest that muscle-targeted therapeutics may be effective in SBMA.

Recent studies have also suggested that muscle may play a more pivotal role in disease pathogenesis than previously thought and, indeed there is evidence that muscle may represent a primary site of AR toxicity (Cortes et al., 2014a; Lieberman et al., 2014; Malena et al., 2013). In a BAC fxAR121Q conditional mouse model of SBMA carrying 121 polyQ repeats within a floxed exon 1 of AR, which enables muscle-specific excision of the expanded CAG repeat by Cre recombinase, a clear improvement in the phenotype was observed in diseased mice (Cortes et al., 2014a). In a separate study, antisense oligonucleotides, which suppressed *AR* gene expression in the muscle but not in the spinal cord, was reported to rescue the manifestation of disease in both the fxAR121Q and AR113Q knock-in mouse models of SBMA (Lieberman et al., 2014). Interestingly, patients generally present with mixed myopathic alterations (centralised nuclei) as well as neurogenic features of denervation (fibre-type grouping, angulated fibres) in muscle biopsy (Soraru et al., 2008), and electromyograms show evidence of denervation atrophy (Breza and Koutsis, 2018; Rhodes et al., 2009). In AR100 mice at 12 months of age, H&E staining demonstrated both neurogenic (fibre type grouping) and myogenic (internalised nuclei) processes, pointing to a mixed-type pathology. Crucially, we show that muscle force dysfunction is the earliest manifestation of disease in AR100 mice, preceding the degeneration of motor neurons.

Muscle force measurements have been performed in SBMA mice previously, both by our lab *in vivo* in AR100 mice (Malik et al., 2013) and also *in vitro* in SBMA mouse models (Oki et al., 2015). Oki et al. performed *in vitro* experiments to show a reduction in muscle force late in the disease process in EDL and soleus muscle in the AR97Q SBMA mice and also using the myogenic mouse model with overexpression of the WT-AR exclusively in skeletal muscle (Oki et al., 2015). We previously studied the neuromuscular deficits in the SBMA AR100 mice at the late stage of disease (18 months), by which point hindlimb muscle atrophy, body weight reduction and neuromuscular dysfunction are extremely pronounced (Malik et al., 2013). Here, we show that, in the AR100 mouse model of SBMA, disease-related changes in muscle force, function and histopathology were absent at an earlier stage of disease (3 months), but by 6 months of age, a clear disease phenotype begins to emerge in fast-twitch (but not slow-twitch) hindlimb muscles. This is a significantly earlier time of symptom onset than previously reported for this model of SBMA (Sopher et al., 2004). TA and EDL muscle force generation, as well as muscle weights, were all significantly reduced in AR100 mice from 6 months of age onwards. Symptom onset in AR100 mice is therefore defined by loss of muscle force generation, which precedes any signs of motor neuron degeneration and changes in overall body weight. Interestingly, the AR113Q knock-in mouse model of SBMA has also been shown to exhibit early signs of myopathy along with signs of neurogenic denervation, before the obvious loss of any motor neurons (Yu et al., 2006).

Significantly, our results also show that the deterioration in the contractile characteristics of TA and EDL occurs primarily in the late stage of disease, and TTP and ½RT were markedly prolonged in AR100 mice by late stage of disease (18 months) relative to WT mice. Although the FI was significantly greater in 6-month-old AR100 mice than WT, this further increased between 12 and 18 months. This change in the fatigue characteristics of these normally fast-twitch (and fast-relaxing) muscles was reflected in an increase in the oxidative capacity of these muscles, with the majority of TA muscle fibres staining strongly for SDH at 18 months, representing a highly oxidative phenotype. These changes in the characteristics of TA and EDL were not observed in the slow-twitch soleus muscle, which was relatively unaffected by disease. The increased vulnerability to disease of fast-twitch TA and EDL in AR100 SBMA mice reflects a similar pattern of selective vulnerability in amyotrophic lateral sclerosis (ALS), a very rapidly progressing MND, in which deficits in fast-twitch muscles manifest very early in disease progression, whereas the slow-twitch soleus muscle remains relatively resistant (Kalmar et al., 2012, 2008; Kieran et al., 2004). The only significant reduction in soleus muscle weights were observed at 18 months, at which point body weight differences between WT and AR100 mice were also considerable.

Thus, our results show that fast-twitch muscles are preferentially affected by disease in SBMA mice and may undergo fibre type switching. For example, in 6 and 12 month AR100 mice, a considerable reduction in MyHC expression specific to fast-twitch glycolytic type IIB fibres regulating explosive muscle function was observed. This may reflect a transitional state to type I slow oxidative fibres. In the knock-in AR113Q SBMA mice, a progressive shift from glycolytic to oxidative fibres has also been reported (Rocchi et al., 2016). Furthermore, SBMA patients also exhibit switching in muscle fibre type from glycolytic to oxidative fibres (Yamada et al., 2016). In addition, as early as 6 months of age, ubiquitin inclusions were observed, predominantly within very fast-twitch type IIB TA and EDL muscle fibres. Although the presence of ubiquitinated inclusions within fast-twitch type IIB fibres is substantial, these do not increase between 12 and 18 months, and there is little further decline in TA and EDL muscle weights during this timeframe. These results show that the deficits in TA and EDL muscle force and weight in AR100 mice are maximal by 12 months of age, with little significant decline thereafter. As motor neuron loss only occurs between 12 and 18 months, our results suggest that the major deficits observed in hindlimb fast-twitch muscles may not be due to motor neuron degeneration, and rather reflect a primary muscle-specific deficit induced by the presence of the expanded mutant AR in the muscles themselves.

The SBMA YAC AR100 mice express the human polyQ-AR similar to endogenous AR levels in the mouse and therefore develop a slowly progressive neuromuscular phenotype consisting of early muscle deficits and later motor neuron loss. In this manner it faithfully recapitulates the course of the disease and many of the features seen in SBMA patients. Pathology first occurs in hindlimb fast-twitch muscle preceding any signs of motor neuron degeneration, which is only evident at later stages of the disease. Therefore, the SBMA AR100 mice display the full range of features of the disease seen in patients, such as muscle atrophy, motor neuron loss and a normal life span, which replicates a characteristic observed in the majority of individuals with SBMA who have a normal life expectancy (Atsuta et al., 2006; Rhodes et al., 2009). These features make the SBMA AR100 mice an ideal model for preclinical testing of therapeutic strategies. Other models recapitulate only some features but neglect pathological hallmarks of the disease such as motor neuron degeneration (Katsuno et al., 2002; Palazzolo et al., 2009; Rusmini et al., 2015; Yu et al., 2006). Furthermore, several transgenic SBMA models result in premature and accelerated death in mice (Badders et al., 2018; Chevalier-Larsen et al., 2004; Katsuno et al., 2002). Importantly, we have used the AR100 model previously in a successful preclinical testing of therapeutics to ameliorate the neuromuscular phenotype in the AR100 mice by affecting loss of motor neurons as well as reducing the muscle force and contractile deficits (tetanic and twitch force, motor unit number and fatigue characteristics) (Malik et al., 2013).

This study set out to examine the progression of the neuromuscular phenotype in AR100 mice and establish the first signs of pathology so as to appropriately target treatments to vital stages of disease. *In vivo* muscle isometric force measurement was used to examine maximum twitch or tetanic muscle force in hindlimb TA and EDL muscle. An early but progressive reduction was found in maximal twitch and tetanic muscle force of TA and EDL hindlimb muscle of AR100 mice. EDL muscle in AR100 mice were less fatigable than control mice and mice became progressively more resistant to fatigue. The results show that most important physiological parameters to analyse in preclinical therapeutic testing would be, in particular, tetanic and twitch force, which may serve as a reliable indicator of drug efficacy. The motor unit number and fatigability may also serve as useful indicators to examine. However, contractile characteristics of the TA as well as EDL (TTP and ½RT) may be only be useful at later stages of disease. However, it is important to note the obvious differences in physiology between humans and rodents, so the findings from the AR100 mice may not be applicable or translatable to human SBMA patients. Interestingly, in SBMA patients muscle contractility was reduced by 22-39% when investigating the specific muscle force (the muscle strength per cross-sectional area) and the muscle contractility (the muscle strength per fat-free contractile cross-sectional area) (Dahlqvist et al., 2019). Furthermore, motor unit number estimation was found to be decreased by nearly 50% in SBMA patients compared to healthy controls (Rhodes et al., 2009). Therefore, these parameters may be useful to test in mice as they may be relevant for the disease.

Although motor neuron degeneration occurs well after the first signs of muscle force deficits, it is important to acknowledge the possibility that motor neuron dysfunction may occur without degeneration (Katsuno et al., 2004) and there also may be neuromuscular junction (NMJ) abnormalities (Xu et al., 2016). Late motor neuron degeneration or dysfunction may well be specific to the AR100 mice and the possibility exists that it may not reflect the scenario in SBMA patients. It is, however, important to recognise that the knock-in AR113Q mice show signs of primary muscle pathology before motor neuron dysfunction (Yu et al., 2006), as do the AR112Q mice (Chevalier-Larsen et al., 2004) and the AR121Q model (Badders et al., 2018). In addition, the loss of motor neurons from the ventral horn of the spinal cord is a characteristic hallmark found in SBMA patients (Kennedy et al., 1968; Lieberman and Robins, 2008). There was no evidence of morphological denervation at the NMJ at 6 months of age in AR100 mice, the age at which muscle force dysfunction first becomes evident. However, there may be functional denervation without morphological changes at the NMJ (Poort et al., 2016; Xu et al., 2016). Although we have shown early dysfunction in motor neurons in AR100 mice, such as ER stress (Montague et al., 2014) and transcriptional dysregulation (Malik et al., 2019), we found that plasma levels of phosphorylated neurofilament heavy chain (pNfH), a marker of neuronal damage found to be altered in motor neuron diseases such as ALS, was unchanged in SBMA patients and AR100 mice (Lombardi et al., 2019). Interestingly, although a marker of neuronal damage, neurofilament heavy chain (NfL), was unchanged in blood samples from SBMA patients and in the AR100 mouse model, muscle damage markers were significantly altered (Lombardi et al., 2019). As axonal transport dysfunction is considered to be linked with synapse function and accumulation and neurofilament levels associated with neuronal damage, the physiology muscle force experiments results show that polyQ-AR may cause early muscle dysfunction in AR100 mice, which may occur independently from loss or injury of motor neurons.

In this study, we show that in the AR100 mouse model of SBMA, the disease first manifests in skeletal muscle in young mice, before any motor neuron degeneration, which only occurs in late stage disease. Our results show that the SBMA mice exhibit a clear progressive neuromuscular decline as they age, with reduced hindlimb muscle force and changes in the phenotype of fast-twitch muscles, followed by the onset of motor neuron degeneration between 12 and 18 months of age, a later stage of disease. The first neuromuscular signs of disease are first observed in fast-twitch muscles at 6 months of age, and these deficits reach a maximum by 12 months, after which there is very little further decline in the muscle phenotype. Motor neuron degeneration is a late manifestation of disease in AR100 mice, and occurs after the decline in muscle function. Our results therefore show that muscle is a primary site of AR toxicity in SBMA, and suggest that targeting muscle deficits may be an effective therapeutic strategy for the treatment of the disease.

## MATERIALS AND METHODS

### Breeding and maintenance of SBMA mice

Mice were bred and maintained at the UCL Queen Square Institute of Neurology Biological Services (London, UK). All experimental procedures were carried out following approval by the UCL Institute of Neurology Animal Welfare Ethical Review Panel and under licence from the UK Home Office (Scientific Procedures Act 1986). Male mice carrying the AR with 100 (pathogenic AR100 mice) or 20 (non-pathogenic AR20 mice) polyQ repeats were mated with WT C57BL/6J females. Genotyping by PCR was performed as described (Malik et al., 2013). Only male mice were used in this study in agreement with the gender specificity of the human disease. Experiments were designed to use the minimum number of animals required for reliable statistical analysis and the 3R principles were considered at each stage of the experimental planning. Mice were housed in controlled temperature and humidity conditions with access to food and drinking water *ad libitum*, and were maintained on a 12 h light/dark cycle.

### *In vivo* analysis of isometric muscle force and motor unit survival

*In vivo* assessment of muscle function was carried out as previously described (Kieran et al., 2004; Kalmar et al., 2008; Malik et al., 2013). The mice were deeply anaesthetised using a Fortec vaporiser (Vet Tech Solutions) by placing the animals in an induction chamber containing 1.5-2.0% isoflurane in oxygen. Anaesthesia was maintained with the same mixture delivered through a facemask. The distal tendons of the TA and EDL muscles in both hindlimbs were dissected free and attached to isometric force transducers (Dynamometer UFI Devices). The sciatic nerve was exposed and all branches cut except for the deep peroneal nerve that innervates the TA and EDL muscles. Isometric contractions were elicited by stimulating the nerve to TA and EDL using square-wave pulses of 0.02 ms duration at supra-maximal intensity. Contractions were elicited by trains of stimuli at frequencies of 40, 80 and 100 Hz for 450 ms and the maximum twitch and tetanic tension was measured using the force transducers connected to a Picoscope 3423 oscilloscope (Pico Technology) and analysed using Picoscope Software v5.16.2 (Pico Technology). The contractile and fatigue characteristics of EDL were also determined. The TTP contraction was calculated by measuring the time taken (ms) for the muscles to elicit peak twitch tension, and the ½RT was the period taken for the muscles to reach half relaxation from peak contraction.

The number of motor units innervating the EDL muscle in both hindlimbs was determined by stimulating the motor nerve with stimuli of increasing intensity, resulting in stepwise increments in twitch tension due to successive recruitment of motor axons. The number of stepwise increments was taken to be the estimate of the number of functional motor units present in each EDL muscle. The resistance of the EDL muscle to fatigue was assessed by repeated stimulation at 40 Hz for 250 ms/s for 3 min. The tetanic contractions were recorded on a Lectromed Multitrace 2 recorder. The decrease in tension after 3 min of stimulation was measured and an FI was calculated, expressed as a percentage of the starting tetanic tension.

### Tissue harvesting

Upon completion of the physiology experiments, the mice were terminally anaesthetised by intraperitoneal injection of 4% chloral hydrate. The TA, EDL and soleus muscles were removed, mounted using optimal cutting temperature (OCT) cryoprotective mounting medium, snap frozen in isopentane and then cooled in liquid nitrogen. Tissues were stored at −80°C until further processing. Muscles were weighed before freezing. Following removal of muscles, mice were transcardially perfused using 0.9% saline followed by 4% paraformaldehyde. The spinal cord was removed and postfixed and then stored in 30% sucrose in PBS at 4°C.

### Assessment of motor neuron survival

Motor neuron counts were performed from within the sciatic motor pool of the lumbar spinal cord as described (Kieran and Greensmith, 2004; Malik et al., 2011; Sharp et al., 2005). Serial transverse 20 μm sections were stained with gallocyanin. Sections were rinsed in tap water followed by dehydration via immersion in increasing concentrations of ethanol. Sections were cleared in histoclear and coverslips were mounted onto the slides using DPX. Motor neuron survival was assessed by counting all large polygonal motor neurons with a clearly visible nucleus and nucleolus. Every third lumbar spinal cord section was counted, to avoid counting any motor neuron more than once. For each animal, 40 spinal cord sections were assessed. The counting of motor neuron number was performed under blinded conditions.

### Muscle histochemistry

Transverse TA 12 μm muscle cryosections were stained for SDH as previously described (Bros-Facer et al., 2014; Kieran and Greensmith, 2004). Briefly, sections were treated with working solution [0.1 M phosphate buffer (pH 7.6), 1 M sodium succinate, 15 mM nitroblue tetrazolium, 0.1 M potassium cyanide and 10 mM phenazine methosulphate] for 5 min at 37°C. Subsequently, sections were rinsed in 0.9% saline followed by rinses in 70% acetone, 90% acetone and 100% ethanol. Sections were immersed in each solution for 1 min followed by two 2 min rinses in histoclear. Coverslips were mounted onto slides using DPX mounting media. Sections were also stained with H&E. In brief, sections were stained in Harris Haematoxylin solution for 8 min, and then rinsed in running tap water for 5 min. Sections were differentiated by immersion in 1% acid alcohol for 30 s and washed in running tap water for 1 min, followed by immersion in 0.2% ammonia water for 30 s. Sections were then rinsed in running tap water for 5 min and rinsed in 95% ethanol. Sections were then counterstained in Eosin solution for 2 min and dehydrated in graded ethanol solutions (70%, 90% and two immersions in 100% ethanol) for 1 min each. The counting of muscle fibre size and number was performed under blinded conditions

### Muscle immunofluorescence

Sections were incubated for 1 h in blocking solution containing PBS 0.1% Triton X-100 (v/v) and 5% serum (v/v) and incubated overnight in the primary antibody in 2% blocking solution. The following antibodies were used: ubiquitin (Dako), Nogo-A (R&D Systems), myosin heavy chain subtypes to identify the combination of muscle fibre type (Developmental Studies Hybridoma Bank: myosin heavy chain 2A BF-F3-s, or myosin heavy chain 2B SC-71-s) (see Table S1 for full details of primary antibodies). Primary antibodies were detected using Alexa Fluor 488 or Alexa Fluor 568 secondary antibodies (see Table S1 for full details) and nuclei were stained with DAPI (Sigma-Aldrich). Images were acquired using a Leica DMR fluorescence microscope.

EDL was stained with α-Bungarotoxin (Sigma-Aldrich, T0195) which labels the postsynaptic acetyl choline receptor, and then stained with ChAT antibody (Millipore), a marker of motor axons (see Table S1). Analysis of innervation was performed as follows: percentage of end plates fully innervated; percentage of end plates partially innervated; percentage of end plates completely denervated.

### Statistical analysis

Statistical analysis was performed using SPSS v23 (SPSS Inc.), with the unpaired Student's *t*-test (two-tailed) or one-way ANOVA with corresponding post hoc tests performed to determine significance of data (*P*<0.05). For non-parametric data the Mann–Whitney *U*-test or Kruskal–Wallis tests were used to establish significance of data.
